# CI-SpliceAI—Improving machine learning predictions of disease causing splicing variants using curated alternative splice sites

**DOI:** 10.1371/journal.pone.0269159

**Published:** 2022-06-03

**Authors:** Yaron Strauch, Jenny Lord, Mahesan Niranjan, Diana Baralle

**Affiliations:** 1 School of Human Development and Health, Faculty of Medicine, University of Southampton, Hampshire, United Kingdom; 2 Vision, Learning and Control, Department of Electronics and Computer Science, Faculty of Engineering and Physical Sciences, University of Southampton, Hampshire, United Kingdom; University of Toronto, CANADA

## Abstract

**Background:**

It is estimated that up to 50% of all disease causing variants disrupt splicing. Due to its complexity, our ability to predict which variants disrupt splicing is limited, meaning missed diagnoses for patients. The emergence of machine learning for targeted medicine holds great potential to improve prediction of splice disrupting variants. The recently published SpliceAI algorithm utilises deep neural networks and has been reported to have a greater accuracy than other commonly used methods.

**Methods and findings:**

The original SpliceAI was trained on splice sites included in primary isoforms combined with novel junctions observed in GTEx data, which might introduce noise and de-correlate the machine learning input with its output. Limiting the data to only validated and manual annotated primary and alternatively spliced GENCODE sites in training may improve predictive abilities. All of these gene isoforms were *collapsed* (aggregated into one pseudo-isoform) and the SpliceAI architecture was retrained (CI-SpliceAI). Predictive performance on a newly curated dataset of 1,316 functionally validated variants from the literature was compared with the original SpliceAI, alongside MMSplice, MaxEntScan, and SQUIRLS. Both SpliceAI algorithms outperformed the other methods, with the original SpliceAI achieving an accuracy of ∼91%, and CI-SpliceAI showing an improvement at ∼92% overall. Predictive accuracy increased in the majority of curated variants.

**Conclusions:**

We show that including only manually annotated alternatively spliced sites in training data improves prediction of clinically relevant variants, and highlight avenues for further performance improvements.

## Introduction

**Fig 1:**
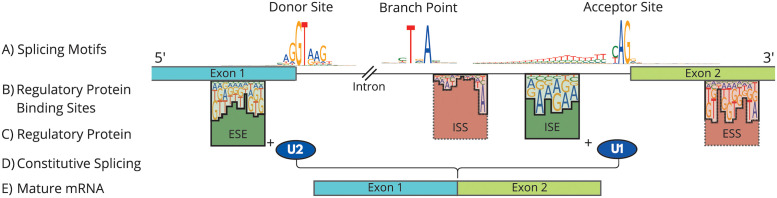
Schematic diagram of splicing process and major regulatory elements. (A) Frequency diagrams at splice sites reveal nucleotide sequence motifs for the acceptor and donor site. The branch point is located upstream of the acceptor. (B) Regulatory protein binding sites can occur anywhere around a splice site. (C) Regulatory protein bind to the motifs and excite or inhibit splicing. (D) The U1 and U2 snRNPs bind to the two splice sites and are attracted to or repelled by regulatory protein. The two junctions are joined to build (E) the mature mRNA that codes for a specific transcript. Branch point and protein binding motifs taken from [4–8].

Splicing is a complex biological process which removes introns and combines exons to produce protein coding transcripts (Fig 1). It is mediated by the *spliceosome*, and regulated by numerous cis- and trans- acting factors, including the splice donor and acceptor sites encoded in the RNA itself [1]. Over 90% of multi-exon genes are believed to undergo alternative splicing, where the same gene can encode multiple different *isoforms* and therefore proteins [2, 3].

Disruption of splicing is a significant contributor to many disorders, from rare diseases to cancer. It is estimated that a substantial amount of pathogenic single-nucleotide variants disrupt splicing [9, 10]. While the exact number is hard to quantify due to bioinformatic pipelines often filtering out synonymous and intronic variants [11], recent studies have shown an improved diagnostic yield of ∼35% through inclusion of RNA sequencing [12–14]. Because of its complexity, predicting which variants disrupt splicing is difficult computationally, leading to a reliance on expensive and time consuming experimental methods to confirm variant pathogenicity. Accurate *in silico* methods would reduce costs and increase speed in a clinical setting.

Many splice prediction tools exist, but there is little consensus on which the best tools are, what thresholds should be used to detect significant disruptions, and how these applications should be applied in clinical diagnostics. Recent applications of machine learning to splice site prediction show great promise, and have been found to be more accurate than older but still widely used methods such as MaxEntScan (MES) [15–17]. MES models the likelihood of a splice site given 9 or 23 bases using a maximum entropy model; *MMSplice* [18] uses neural networks to predict splice sites given 18 or 53 nucleotides; *SQUIRLS* [19] uses carefully engineered features from around the splice sites to classify using decision trees; and *SpliceAI* [20] uses five deep convolutional neural networks to predict splice sites based on 10,000 nucleotides of context. SpliceAI models the splicing process directly on a per-nucleotide basis, which may lead to insights into the splicing mechanism itself.

**Fig 2:**
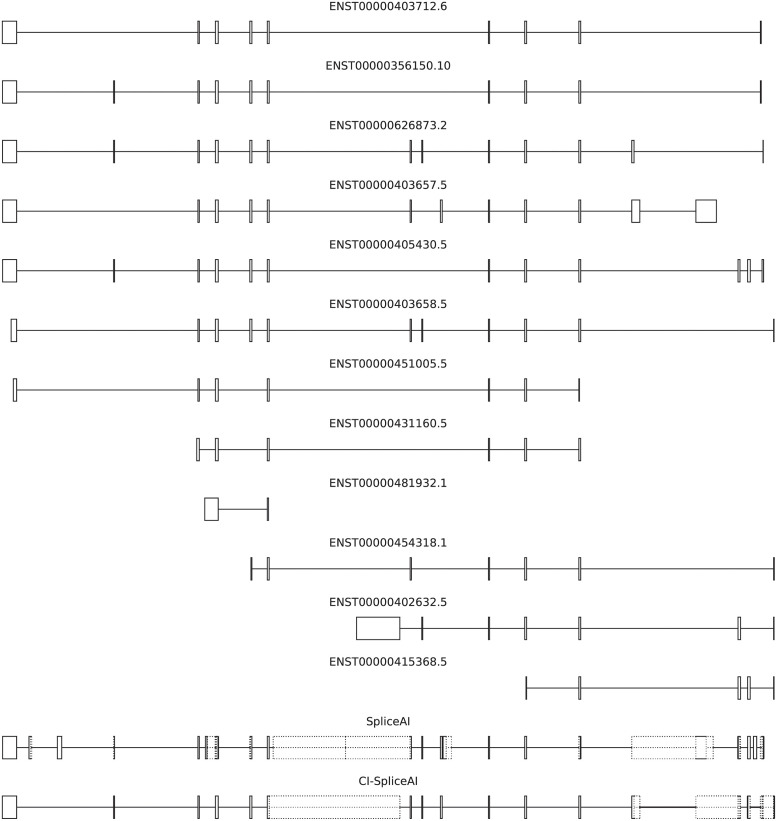
All manually annotated and validated *SH3YL1* isoforms on GENCODE v37GRCh38, and the training data used for SpliceAI and CI-SpliceAI. SH3YL1 is the first gene on the SpliceAI training data with more than one validated transcript. Ambivalent regions that can code for both exons and introns are depicted by dotted lines. On this gene, 26 (46%) of splice sites in the SpliceAI training data are not in HAVANA; 7 (18%) of our splice sites are not present in SpliceAI training data.

The original SpliceAI algorithm was trained on *GENCODE* v24GRCh37 [21] data, using a single selected primary isoform per gene, enriched with “novel splice junctions commonly observed in the GTEx cohort” [20]. The human reference genome in version GRCh37.p5 [22] provided sequencing input. This means many validated alternative transcripts from GENCODE are not included in the training data, and that unchecked and novel splice sites from GTEx are. GTEx splice sites not contained within GENCODE may also result from changes in GTEx participant DNA not present in the reference genome that SpliceAI is trained on, decoupling the relationship between DNA sequence input and splicing outcome. To overcome both issues, we retrained the model using a *collapsed* isoform set representative of all manually annotated constitutive and alternative splice sites from GENCODE (Fig 2), also known as HAVANA annotations.

We compare the performance of this *Collapsed Isoform SpliceAI* (CI-SpliceAI) algorithm against the original SpliceAI, MMSplice, SQUIRLS and MES using a set of 1,316 variants functionally tested for splicing impact from the literature. The best thresholds on this data are determined empirically to assist future similar studies. Comparing CI-SpliceAI to SpliceAI, we report gains in overall accuracy, improvements of scores for the majority of variants tested, and higher quality of variant effect predictions. We demonstrate modifications in the data pipeline can improve performance, enhance clinical utility, and highlight areas for further refinement.

## Methods

### Splice site recognition

The final SpliceAI algorithm is trained on 19 chromosomes (excluding 1,3,5,7, and 9). We trained our model twice: 1) on the 19 training chromosomes to allow a fair comparison to the original SpliceAI algorithm; and 2) on the entire dataset which is used as our final predictive model. The inclusion of all chromosomes should prevent biasing the model against those genes held out in the train set.

The model from 1) was then tested on the remainder of chromosomes excluding paralogs, which were taken from Ensembl BioMart.

GENCODE annotations (v37GRCh38) [21] were filtered to exclude level 3 transcripts (automated annotation), so all training data was annotated by a human. Exon annotations were matched to their transcripts by transcript ID. Exon boundaries that aligned with a transcript start or end annotation were removed, implicitly removing transcripts/genes consisting of only one exon. All remaining splice sites for all transcripts per gene were aggregated into one collapsed representation.

Each transcript region was enlarged by 5,000 nucleotides on both sides to provide 10,000 nucleotides of context, and sequence data was extracted from GRCh38.p13 [23]. Sequences were sliced into windows of 15,000 bases, where each slice predicts 5,000 nucleotides at a time given 10,000 bases of flanking context. Genes on the negative strand were reverse-complemented, short slices were padded with ‘N’ (*unknown nucleotide*) if necessary. Both the sequence data and the 5,000 ground truth positions (acceptor/donor/neither) were one-hot encoded.

The training process was reimplemented from SpliceAI [20]. The training data was aggregated into chunks of 100 genes (the last two chunks being combined so no chunk has less than 100 genes). Each of the five models is trained on two GPUs given a random subset of 90% of chunks, with the remainder functioning as validation data. They are trained for ten times the number of train chunks by randomly selecting one chunk and iterating over it gene by gene, and descending the gradient of the categorical cross-entropy error using Adaptive Moment Estimation (Adam) after each gene. The initial learning rate of 0.001 is halved after 60%, 70%, 80%, and 90% of the total train process.

### Curated variant data

Variant data was aggregated from the literature [24–29]. Erroneous *Human Genome Variant Society IDs* (HGVS-IDs) [30], such as incorrect annotations on reverse-stranded genes or outdated transcript identifiers, were adjusted when needed. GRCh38 coordinates were queried from their HGVS-IDs using *ensembl VEP* [31]. Duplicates were resolved based on their genomic location. Details about aggregation and manual data correction can be found in S2 Appendix in S1 File.

Specific variant effects, such as which specific exon is skipped or which novel/alternative splice site is used, were parsed manually where available, allowing investigation into prediction accuracy on exact variant effect.

### Algorithmic comparison on variant data

#### Algorithms

All five algorithms (MES, MMSplice, SQUIRLS, SpliceAI and CI-SpliceAI) were assessed on this curated variant set.

MES [15] was run in two different ways. 1) As an Ensembl VEP [32] plugin [33], which checks for donor and acceptor sites within 9 or 23 bases from the variant respectively to calculate one reference and one variant score, which will disregard all cryptic sites. And 2) through application as a sliding window (similar to [19]) around each variant in a custom python implementation, enabling detection of newly generated cryptic splice sites.

MMSplice [18] was run through *kipoi* [34], a python manager for genomic models. The two most appropriate MMSplice models, namely *splicing efficiency* and *pathogenicity*, were run on a *variant call format* (VCF) file which was normalised and left aligned.

SQUIRLS was run on the variant VCF file as described in [19].

Both SpliceAI [20] and CI-SpliceAI were run using their respective published python VCF annotation module, with a maximum distance from the variant set to 5,000. For SpliceAI, we configured 4,999 nucleotides max-distance as the python library does not support more than that nucleotides to account for deep variants. Both implementations extract the reference sequence plus the maximum distance from a variant and a further 5,000 nucleotides providing context on either side, one-hot encode reference and variant sequences, and run them through their respective neural networks. They both compensate insertions and deletions by padding variant predictions with zeros for deletions, or using the max function for insertions, ensuring alignment with the reference coordinates (more details in S3 Appendix in S1 File). Our VCF annotation library follows [35] with some optimisations such as allowing to process multi-nucleotide indels (such as GTT>AA). Our VCF annotation module and its application can be found through our portal (S1 Application, Data, Code in S1 File).

For the two SpliceAI models and MES, the *delta score* (difference between reference and alternative sequence scores) was derived. If the maximum absolute delta score exceeds the defined threshold at any nucleotide, the variant is determined to be splice affecting. The SpliceAI authors used a main threshold of 0.2 and recommended higher thresholds for more specificity [20].

For every tool, we report the optimal threshold in our dataset, i.e. the threshold with maximum accuracy across all possible thresholds. On this data, the *percentage deviation* (i.e. [36, 37]) decreased predictive performance for all tools and was omitted.

#### Variant experiment designs

Algorithm performance was assessed in two different ways: 1) the *binary classification task* which evaluates how well the algorithms predict if a variant affects splicing or not (all algorithms, full data set, missing predictions are filled as non-affecting); and 2) the *exact classification task* investigating how well the exact position and effect of the variant on splicing could be determined. The second experiment could only be conducted on algorithms that predict scores per offset (SpliceAI, CI-SpliceAI, and the sliding window MES), and on variants where the exact variant effect was known.

## Results

### Training data

The collapsed isoform training set contains more splice sites, genes, and chromosomes than the original SpliceAI training data (Table 1). When filtered to the same chromosomes, the number of genes in CI-SpliceAI is slightly smaller due to the newer GENCODE version containing fewer transcripts of low quality. 18% of start and stop annotations of genes (primary transcript for SpliceAI, collapsed isoform for CI-SpliceAI) and 58% of splice sites overlap between the lifted SpliceAI training set and ours when filtered to the same chromosomes.

**Table 1:** Numeric comparison between the original SpliceAI training set and our novel collapsed dataset.

	No. Chroms	No. Genes	No. Splice Sites	Proportion Acceptor/Donor
**SpliceAI (Train)**	19	13,385	391,515	2.1% more acceptor sites
**CI-SpliceAI (Train)**	19	13,240	301,835	3.5% more donor sites
**CI-SpliceAI (All)**	24	18,580	428,475	3.4% more donor sites

The novel collapsed training dataset used for training (bottom row) includes all chromosome, more genes, and more splice sites than SpliceAI. For comparison only, if filtered to the same chromosomes as SpliceAI (middle row), the collapsed dataset would have slightly fewer genes and splice sites than SpliceAI, due to newer GENCODE versions and filtering. While the SpliceAI dataset has slightly more acceptor than donor sites, ours has slightly more donor sites.

### Splice site recognition

The five neural networks trained on 19 chromosomes can predict splice sites on the remainder (excluding paralogs) with 94% area under the PR curve, less than the 98% when trained on primary isoforms [20, Fig 1E] and more than the 84% on the SpliceAI GENCODE+GTEx dataset [38].

**Fig 3:**
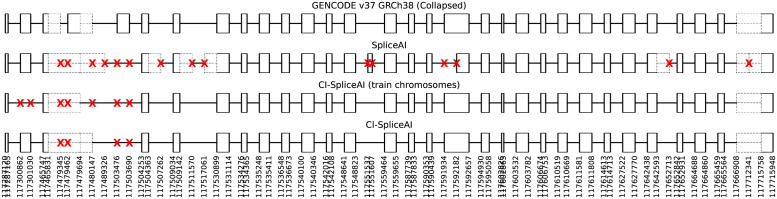
Predictions of splice sites in CFTR in comparison to our ground truth from GENCODE. Mispredictions are marked with a red X. The original SpliceAI algorithm misses one exon, adds one extra, and mispredicts 15 sites in total. When trained on collapsed the curated GENCODE sites (train chromosomes), CI-SpliceAI misses two exons, does not add any extra exons, and mispredicts 7 sites in total. When trained on the collapsed isoform data and all chromosomes (which includes this gene), one missing exon is detected correctly and the overall error decreases to 4 mispredicted sites in total.

We used both SpliceAI and CI-SpliceAI (trained on the 19 training chromosomes) to predict splice sites on the gene CFTR (comparable to [20], Fig 1B), located on chromosome 7 which is excluded from the training data (Fig 3). While SpliceAI has 15 mispredictions, half of them false positives, CI-SpliceAI has 4 mispredictions, all of them false negatives.

### Variant data

**Fig 4:**
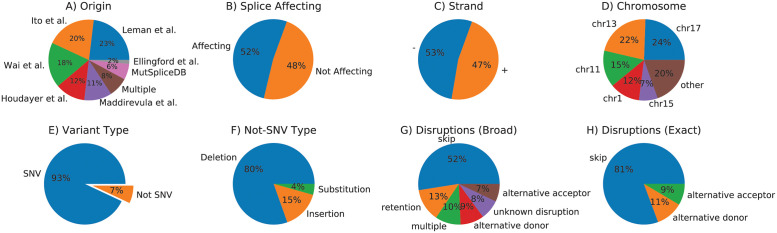
The final curated variant data set consists of 1,316 unique variants. (A) Literature sources of our data. There is an overlap of 8% of variants (mainly due to Houdayer et al. citing Leman et al.), all with consensus. (B) Split between splice affecting and non-affecting variants. (C) Split between negatively and positively stranded genes. (D) Variant location on chromosomes. Chromosomes 1, 11, 13, and 17 make up 73% of the variants. (E) Types of variants. (F) Split of non-SNV variant classes (7% of the whole data). (G) Functional impact of splicing variants. (H) Type of disruption for the 388 variants where the exact genomic location of the disruption is known (mostly exon skipping, with 19% using a specific alternative acceptor or donor site).

1,316 unique variants with functionally validated splicing impact were sourced from the literature (see S2 Appendix in S1 File). Fig 4 shows that the variants are reasonably balanced between splice affecting and non-affecting variants as well as the strandedness, preventing some potential biases, however due many of the publications investigating breast cancer, the chromosomes are biased towards chr17 (*BRCA1*) and chr13 (*BRCA2*). The vast majority of variants are SNVs, with deletions next most common. 388 variants have exact genomic coordinate annotations that describe the effect, i.e. the genomic coordinates of a loss or gain event, so that algorithmic effect outputs can be compared more granularly.

**Fig 5:**
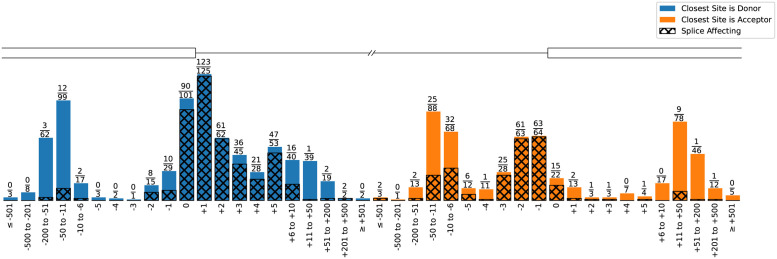
All 1,316 variants in relation to their closest splice site and if they affect splicing (shaded area and numerator in the fraction). 57% of sites are closer to a donor than an acceptor site. 23% of variants are in the consensus motif (equally split between acceptors and donors); 98% of variants within consensus regions are splice affecting.

Fig 5 shows the variant distribution relative to their closest splice site. As expected, variants nearer to a splice site are more frequently disruptive. The dataset includes some deep intronic variants that affect splicing and variants near splice sites that do not, both of which are challenging to predict.

### Algorithmic comparison on variant data

Five splicing prediction algorithms were run on the 1,316 splice tested variants described above. Table 2 shows the area under the precision-recall and receiver-operater characteristic curve, the optimal threshold found on the data, its corresponding accuracy, and the coverage of how many variants have been annotated for all algorithms discussed. The optimal thresholds were empirically calculated to give the greatest accuracy on the large, curated variant set. The two SpliceAI models outperform the others, with CI-SpliceAI showing improved performance over SpliceAI by around one percentage point on all measures and an accuracy of over 92%. The SpliceAI module misses three annotations for which its lookup table contains no transcripts in that region and seven variants are not annotated because REF and ALT sequences are both longer than one basepair. CI-SpliceAI annotates all variants and therefore reaches 100% coverage, together with SQUIRLS and the sliding window MES. While MES, as run through VEP, provides scores for only 58% of all variants (missing 553 variants where no overlapping introns were found), its accuracy (>86%) is considerably higher than when applied as a sliding window (>53%) which in turn provides annotation of all variants. The pathogenicity model of MMSplice outperforms their splicing efficiency model by a percentage point. Both models miss 8 predictions, all of which are deep intronic (>100bp).

**Table 2:** Binary classification results on the variant data (affecting/non affecting).

	Coverage	AUC-PR	AUC-ROC	Optimal Threshold	Accuracy
**MES (Sliding Window)**	**100%**	55.68%	52.97%	12.500	53.42%
**SQUIRLS**	**100%**	91.32%	91.17%	0.074	85.64%
**MES (VEP)**	58%	92.52%	89.15%	2.109	86.40%
**MMSplice (Splicing Efficiency**)	99%	93.03%	92.56%	1.119	87.23%
**MMSplice (Pathogenicity)**	99%	94.13%	92.84%	0.961	88.53%
**SpliceAI**	99%	96.21%	95.65%	0.300	90.88%
**CI-SpliceAI**	**100%**	**97.25%**	**96.75%**	0.190	**92.17%**

Comparison of coverage (i.e. how many variants were annotated by the algorithm), the area under the precision-recall (PR) and receiver-operator characteristic (ROC) curve, the optimal threshold found, and the accuracy on this threshold for the different algorithms ran on our dataset. This is measured on all 1,316 variants.

**Fig 6:**
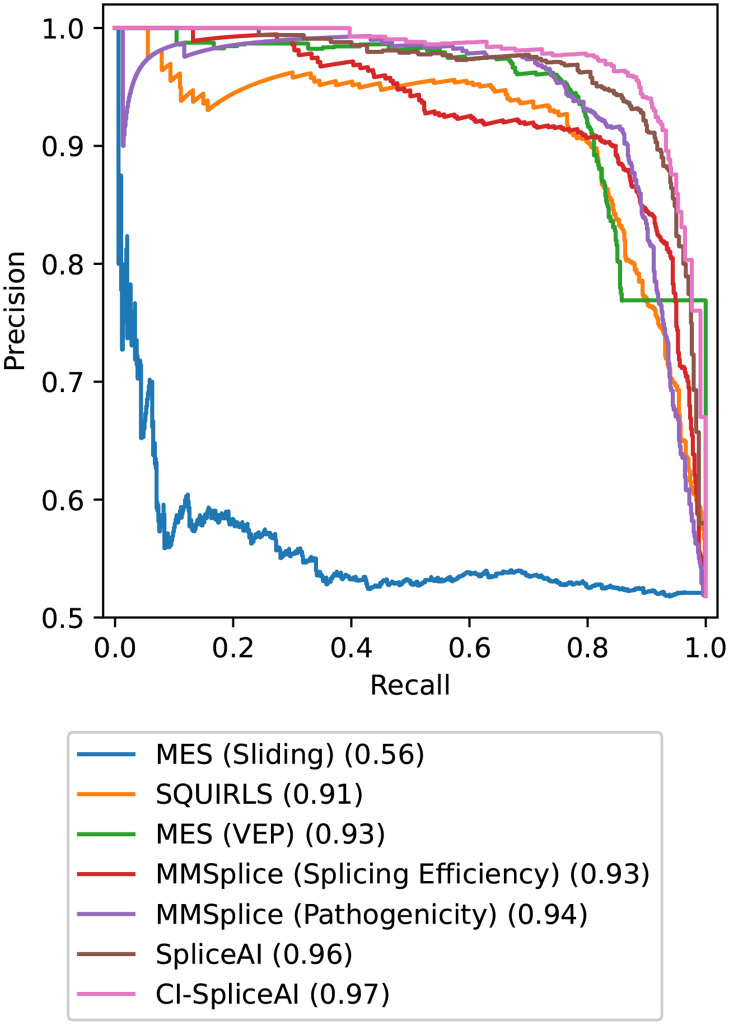
PR curves of algorithms investigated on all 1,316 variants. PR curves demonstrate the capabilities of a model to trade off precision and recall. The area under each curve is indicated in the legend.

The precision-recall curves are visualised in Fig 6. The smooth shapes of the SpliceAI and CI-SpliceAI curves illustrate good clinical application due to few fluctuations in predictive performance when selecting for a desired precision/recall trade-off. Irregular shapes (sliding window MES, VEP MES for high recall, and MMSplice pathogenicity for high precision predictions) indicate problems in the model that limit clinical application.

**Fig 7:**
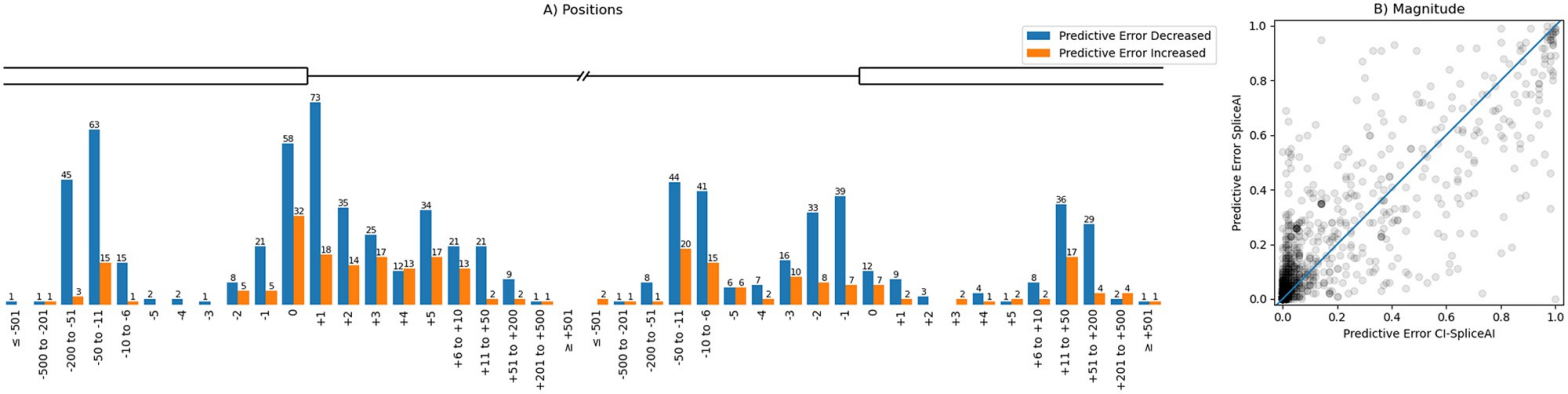
Comparison of the predictive error between SpliceAI and CI-SpliceAI on all 1,316 variants. The predictive error is the absolute difference between the most significant predicted annotation and the ground truth. 78% of predictions in CI-SpliceAI have a different output than when annotated with SpliceAI, out of which 73% have a smaller predictive error. (A) Predictions for CI-SpliceAI improved relative to SpliceAI for almost every position; there is no obvious bias where predictions worsened at a specific distance from a splice site, neither for the type of splice site nor the distance. (B) The magnitude of the change in predictive error. Variants on the identity line did not change their score; points lying above the identity line improved, points below worsened. The big cluster at the bottom left corner represents points where our algorithm shows improved confidence in correct predictions. A cluster of mispredicted variants of high confidence have worsened (top right corner).

When comparing the predictions made by the original SpliceAI vs CI-SpliceAI, the prediction of 78% of variants changed, of which 73% improved (i.e. scores moved towards the results of functional analysis, Fig 7).

In addition to the binary task of predicting whether or not a variant affects splicing, for a subset of 388 variants, the exact location of splicing disruption was known. Table 3 compares the accuracy of predicting the exact genomic coordinates of the variant effect. This is only possible for algorithms that return one score per nucleotide offset (the two SpliceAI algorithms and the sliding window MES). The deep learning models outperform MES, with CI-SpliceAI exceeding or matching SpliceAI’s accuracy on all four effect classes.

**Table 3:** Exact classification results on the curated variant data.

	Acceptor Gain	Acceptor Loss	Donor Gain	Donor Loss
**MES (Sliding Window)**	0.00%	1.16%	2.33%	2.25%
**SpliceAI**	87.50%	77.10%	**79.07%**	78.93%
**CI-SpliceAI**	**93.75%**	**78.55%**	**79.07%**	**82.02%**

Comparison of the accuracy of predicting the exact location of the splice disruption on the 388 variants with known exact disruption coordinates. Except for donor gains, where CI-SpliceAI and the original SpliceAI have equal accuracy, CI-SpliceAI outperforms other models.

### Application

We release CI-SpliceAI with all its components open source on our portal (S1 Application, Data, Code in S1 File). The portal also allows annotation of VCF files online for non-commercial use free of charge; these variants are processed on the google cloud and cached to a database to prevent redundant computation.

## Discussion

Disruption of splicing is a major contributor to human disease, including rare disorders, cancers and neurodegeneration. The ability to accurately predict the impact a variant will have on the splicing process would improve diagnostics and enhance understanding of disease processes. Despite many *in silico* algorithms for splice prediction being available, there is little consensus on the best tools to use in classifying variants, and the cutoffs to use to gain the greatest accuracy. Here we assessed the predictive performance of several *in silico* approaches on a large curated set of functionally tested splicing variants. We show the greatest accuracy from the deep learning based algorithm SpliceAI can be further improved upon through the use of collapsed isoform training data, and we suggest thresholds to maximise accuracy for all tools (Table 2), guiding clinical implementation.

We make the curated variant set (S2 Appendix in S1 File), all training data, the code comparing all tools, the CI-SpliceAI training code and the command line tool freely available on our portal (S1 Application, Data, Code in S1 File).

The curated set of 1,316 variants derived from the literature is one of the most extensive sets of tested splicing variants to date. On this data, CI-SpliceAI had the greatest accuracy of all tools tested on both the binary task and when predicting the exact variant effect. SpliceAI has performed favourably in many comparisons since its release in 2019 [16, 17, 20, 24, 29], and it was suggested that the simultaneous prediction of thousands of nucleotides around a variant is the key advantage for its success: the big window sizes might allow SpliceAI to recognise pairs of acceptors and donors and other co-dependent features not only near splice sites, but also deep within the exon or intron. Feature maps within a convolutional neural network that recognise patterns in data are applied as a sliding window, allowing splicing factors such as binding sites or the branch point to be recognised independent of their offset to a splice site, and variants within their motifs are considered in the classification. This per-nucleotide modelling of the splicing process also allows more granular predictions of the variant effect on the mRNA.

In this study, we show that modifications to the data used to train SpliceAI, while maintaining the same fundamental architecture, can improve predictive performance of splice site disruptions by over 1 percentage point. As accuracies approach 100%, it is to be expected that performance improvements will be modest and incremental. There are likely several factors responsible for this improved accuracy. Firstly, the original SpliceAI included novel junctions from GTEx data that have not necessarily been verified, whereas CI-SpliceAI included only GENCODE splice sites that have been manually annotated. We argue this filtering increases the quality of training annotations. As the exact GTEx data pipeline used by SpliceAI was not published, analysis of their exact method and filtering is non-trivial. Secondly, by including GTEx specific splicing events but not their respective genomic sequence input, a mismatch may be created between DNA input and splicing output annotations. The algorithm may therefore misattribute splicing events subject to specific GTEx participants to the human reference genome sequence that is not spliced. This would also explain why the PR-AUC of SpliceAI on the shared GENCODE+GTEx data is so low, and why measures on HAVANA annotations improved. Thirdly, we included all chromosomes in our training data, whereas SpliceAI excluded chromosomes 1,3,5,7, and 9. We have verified that this is not the single cause of why CI-SpliceAI is better: When including all chromosomes within the SpliceAI training algorithm, the performance improvement was only 0.05% PR-AUC on the clinical data. Finally, SpliceAI was trained using GENCODE v24 and the GRCh37 reference build, both of which have been superseded by more recent versions. Although newer versions improve accuracy and coverage of both resources [39, 40], and we too observed a higher quality of data, comparisons of models trained on new and old versions revealed differences in accuracy were negligible.

Of the other tools tested, all achieved accuracies >85%, with the exception of the sliding window MES application, which did not perform notably better than random chance. This approach was implemented because MES as run through VEP was only able to provide predictions for ∼60% of variants, limited to variants that occur within the vicinity of known splice sites. These predictions will necessarily miss disrupting deep intronic/exonic variants and those creating alternative splice sites, restricting its clinical utility. While the sliding window approach mitigated this limitation, clinical application is inadvisable due to poor predictive performance. MMSplice and SQUIRLS both gave good coverage of variants (>99%) and had relatively high accuracy (∼ 86 − 88%). Their design however means that neither can predict the actual variant effect and its position. Accurate determination of the precise splicing impact has implications for clinical interpretation, for example, in determining whether the splicing change disrupts the reading frame and is likely to lead to nonsense mediated decay. The design of SpliceAI and CI-SpliceAI models the splicing process on a per-nucleotide basis, which allows predictions of the actual variant effect with high accuracy. CI-SpliceAI outperforms or equals other tools on all measures, again, demonstrating the benefits of the collapsed training set.

Many bioinformatic tools require time and computational expertise to be set-up, especially deep learning tools with dependencies on computational graphic cards. But out of the tools evaluated on this data, deep learning tools are significantly better in predicting splice site disruptions, so efforts to improve and encourage their wide spread use in the clinical setting are certainly warranted. Alamut Visual [41] simplifies access to open source splice prediction software (and more) to a common interface, however it is a commercial tool not accessible in the public domain. Ensembl VEP [32] is an open source tool with a freely accessible web interface that allows third-party tools to be run as a plugin. The web interface supports outputs of pre-computed SpliceAI and MES scores for canonical splice sites. We believe that open source and simplified access is vital to research and clinical application, and therefore release CI-SpliceAI both as an open source tool and through a freely accessible web interface.

Under American College of Medical Genetics (ACMG) guidelines [11], *in silico* methods for splice prediction may be used as supporting evidence only where multiple lines of computational evidence suggest no impact (BP4) or deleterious impact (PP3) on gene function. The results presented here highlight the most appropriate tools to use, and suggest empirically derived thresholds to obtain optimal accuracy.

Although the variant set used to test the algorithms is one of the largest curated to date, it is still limited in size, particularly when assessing the exact variant effects. Most data sources did not report the specific splicing change with base-pair resolution, limiting the number of variants available for this assessment. The variant set is also subject to various biases in ascertainment from the sources used. For example, a large proportion of the variants are derived from papers with a focus on breast cancer, leading to an over-representation of certain chromosomes which harbour the BRCA genes. Other sources pre-filtered variant candidates computationally. This may limit the generalisability of the findings, both for tool performance and optimal thresholds reported, to the wider clinical setting. We have also identified a cluster of high confidence mispredictions (Fig 7 top-right corner) where the inclusion of alternative splice sites increased the scores to be even more confident. This might indicate problems in the variant data that can only be resolved by re-evaluation through functional analysis.

While accuracies >90%, as observed for the SpliceAI models, are impressive, there is still clearly room for improvement before *in silico* predictions can be fully trusted in clinical variant interpretation. While CI-SpliceAI does show improved accuracy over SpliceAI, this improvement is modest, impacting 972 out of 1316 variants (73.86%); the accuracy on the remainder decreased. Naturally, the greater the accuracy of a tool, the smaller the room for improvement. However, this study shows that relatively minor adjustments in model development can make an impact on overall performance. Apart from potential simplifications on the model architecture for faster classification, further data engineering (e.g. incorporation of conservation scores, splicing factor binding sites, tissue specific splice site usage levels) could enhance predictive accuracy and timely diagnosis further.

To our knowledge, this study was the first to investigate computational modelling of the actual variant effect, moving away from a simple binary affecting/non-affecting problem formulation and towards predicting the actual impact of a variant on the mRNA sequence. We encourage future splicing studies to report the exact variant effect at base-pair resolution where possible to allow extending this analysis further. We believe that more granular modelling and investigation of potential biases in both training and variant data will enable even more accurate diagnosis of splicing related disease.

## Conclusion

In summary, we compiled an extensive set of functionally validated splicing variants, and used these to test a variety of *in silico* splice prediction methods. We found SpliceAI outperformed other methods, and that its predictions could be improved upon through the incorporation of alternative splicing sites in training data. For each tool, we calculated optimal thresholds for use in the clinical setting. All data and models are made available to facilitate adoption of the method in clinical practice, and further improvements to model performance.

## Supporting information

S1 File (PDF)

S1 Data The dataset of all 1,316 variants, their functionally validated impact on splicing, literature source, and exact variant effect annotation. (CSV)
