# Supplementary material for: CI-SpliceAI—Improving machine learning predictions of disease causing splicing variants using curated alternative splice sites
Source: PLoS One. 2022 Jun 3;17(6):e0269159. doi: 10.1371/journal.pone.0269159 (PMC9165884; doi:10.1371/journal.pone.0269159)
Supplement: S1 File — (PDF) [file pone.0269159.s001.pdf]

S1 Application, Data, Code. The application CI-SpliceAI (online and offline), the variant dataset, our data pipeline, and our code for training, testing and analysis, can be found on web portal on `https://ci-spliceai.com`.

S2 Appendix. Scraping and Quality Control of Variant Data

Incorporating Wai et al.

Table S1 of [1] consists of 258 (actually 259) variants across 65 genes. There is a duplicate HGVS ID (variants 32/33), where apparently there was a copy-and-paste error. In the original publication, variant 32 was incorrectly called NM_007294.3:c.5024C>T (duplicating the entry below) and with the authors help the ID was corrected to NM_007294.3:c.5074+7C>T. Variant 220 is really two; the authors could not determine which variant was causing the effect, so both variants were removed.

The splicing annotation from the source was changed to a binary form ("Normal"/everything else). After parsing the RefSeq ID to genomic coordinates, 12 variant locations were found to be offset by 1bp. This was rectified by fetching all genomic coordinates (see S2 Appendix).

Incorporating Maddirevula et al.

The publication [2] contains table S1 with an aggregation of 272 (269 really since 3 were not disclosed) variants, 124 new ones, 50 previously published variants across 45 publications, and 98 without attribution.

A number of HGVS IDs were not recognised by Ensembl VEP, so were manually amended. Furthermore, only data points where the RT-PCR outcome indicated a conclusive splicing disruption were included.

NM_001040656.1 was deprecated by NCBI, NM_001077416 is not supported by ensembl VEP, both variants were removed.

Incorporating Leman et al.

Tables S1-S3 from [3] were used, containing a total of 254 variants (141 breast cancer variants of their own, the rest compiled from 66 publications) across 11 genes.

NM_007294.3:c.133_136del is an invalid ID that could not be manually resolved as it's unclear if this is a single nucleotide deletion or if it's removing a range of nucleotides. Transcript/Variant annotations were used to generate HGVS IDs, and the *Splicing_Effect* field was used as ground truth.

Incorporating Houdayer et al.

Houdayer [4] includes 272 variants for *BRCA1* and *BRCA2* and partially overlaps with the Leman [3] dataset.

65 of the variants are published as a HTML table and 207 variants on a PDF table across 17 pages. Annotations from HTML were extracted through copy-and-paste into Microsoft Excel, the PDF table was parsed using Tabula [5], followed by manual correction of OCR issues.

12 annotations where the outcome was not obvious were removed, only retaining entries tagged as acceptor/donor loss/gain / skipping / retention. One variant had no annotated observation (NM_000059.3:c.7056T>A), which was also removed. Some IDs contained recurrence annotations in their ID, which were removed as that was syntactically invalid. Two variants (NM_000059.3:c.7397C>T, NM_007294.2:c.5074+68T>C) had mismatching reference annotations and were removed. NM_007294.3:c.5077_5080del4ins10 has a missing insertion annotation and was removed. NM_000059.3:c.9257-10insT and NM_007294.2:c.5277+48_59dup12 could not be parsed by Ensembl VEP.

When resolving duplicates, it was found that five variants that Leman et al. accredit this paper for, are not actually published by this paper. Where these variants come from could not be determined, some may represent those removed due to incorrect annotations.

Incorporating Ito et al.

[6] published 57 *LMNA* variants in their table S5, 139 *MYBPC3* variants in their table S6, and another 43 and 31 (30 due one duplicate) *LMNA* and *MYBPC3* genes respectively in their table S7. Using splice assays in kidney cells, they compared normal and abnormal splicing reads and their statistical significance.

For *LNMA*, the RefSeq ID NM_170707.4 was used, *MYBPC3* was translated to NM_000256.3. Variants NM_170707.4:c.89C>A, NM_170707.4:c.95C>T, and NM_170707.4:n.890G>T have mismatching reference annotations, likely due to updates of the reference genome. These variants were not splice affecting and were removed.

The remaining 267 variants were extracted. Following their paper, variants with an annotated P-Value < 0.01 were annotated as splice affecting; the remainder as non-affecting.

Incorporating Ellingford et al.

Table 1 of [7] published 21 variants and their functional assessment of splice disruption which have been extracted manually.

Incorporating MutSpliceDB

MutSpliceDB [8] is a freely accessible genome database consisting of, at the time of writing, 86 variants and their effects on splicing. All variants are disruptive. Variants were exported using their web interface.

Extraction of Genomic Coordinates

GRCh38 coordinates for the HGVS IDs were fetched automatically using *ensembl VEP* [9].

Errors returned by this service were resolved manually by querying the RefSeq ID in NCBI *Nucleotide* [10] that will link to the latest RefSeq transcript version. For some IDs this still failed, in which the version was removed completely. If both strategies failed, manual investigation revealed some faults that could be rectified (missing colons, mangled protein annotations, missing right-bounds); the remainder of incorrect HGVS IDs (mismatching reference annotations, deprecated transcripts, missing variant annotations, or ambivalent range annotations) were dropped.

S3 Appendix. Calculation of the Delta Score and indel compensation.

The difference between predictions for canonical and alternative sequences build the δ-score, which in most cases translates into subtracting the two predictive matrices directly, i.e. $\delta = P_v - P_r$. This however does not work for indels, where the shape of the predictive matrices differ and therefore cannot be subtracted directly. Following [11], the variant annotations are changed by either padding deletions or truncating insertions using a max function ($P_{v'}$, Fig 8). This method prevents offsets by aligning indels back to the reference genome.

Given the delta matrix, the delta position (DP) and delta score (DS) for the most significant events for the events acceptor gain (AG), acceptor loss (AL), donor gain (DG) and donor loss (DL) are commonly extracted (see last step in Fig 8). For those data points, where the exact effect and position of the variant is known, the significance and position is compared to the annotations in the *exact classification task*

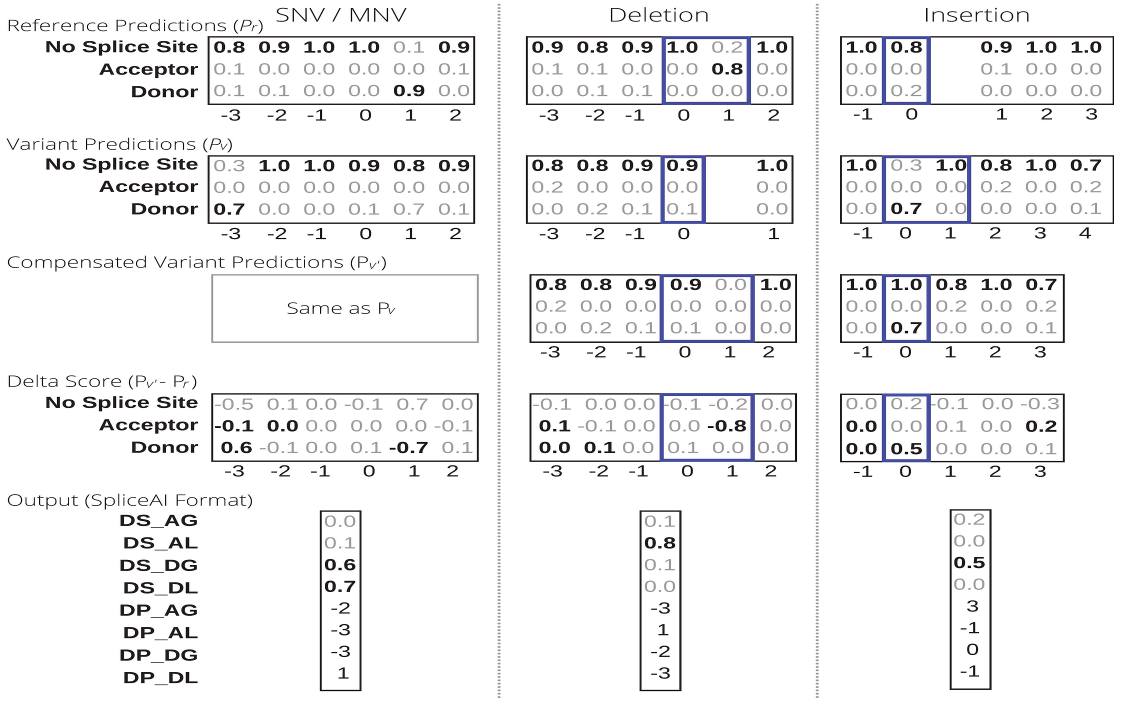

Fig 8. How the delta score is calculated. The blue rectangle indicates an indel event where the output matrices P_r and P_v for reference and variant predictions do not align and need to be compensated. $P_{v'}$ is the re-aligned variant matrix, which either pads deletion predictions with zeros or truncates insertion predictions with a max function. The delta score can then be calculated by subtraction. SpliceAI annotations return the delta score (DS) and delta position (DP) for the maximum and minimum values on the acceptor and donor row, quantifying and locating the events acceptor gain/loss (AG/AL) and donor gain/loss (DG/DL).

References

1. Wai HA, Lord J, Lyon M, Gunning A, Kelly H, Cibin P, et al. Blood RNA analysis can increase clinical diagnostic rate and resolve variants of uncertain significance. Genetics in Medicine. 2020; p. 1–10.
2. Maddirevula S, Kuwahara H, Ewida N, Shamseldin HE, Patel N, Alzahrani F, et al. Analysis of transcript-deleterious variants in Mendelian disorders: implications for RNA-based diagnostics. Genome biology. 2020;21(1):1–21.
3. Leman R, Gaildrat P, Le Gac G, Ka C, Fichou Y, Audrezet MP, et al. Novel diagnostic tool for prediction of variant spliceogenicity derived from a set of 395 combined in silico/in vitro studies: an international collaborative effort. Nucleic acids research. 2018;46(15):7913–7923.
4. Houdayer C, Caux-Moncoutier V, Krieger S, Barrois M, Bonnet F, Bourdon V, et al. Guidelines for splicing analysis in molecular diagnosis derived from a set of 327 combined in silico/in vitro studies on BRCA1 and BRCA2 variants. Human mutation. 2012;33(8):1228–1238.
5. Tabula. Tabula: Extract Tables from PDF; 2018. Available from: `https://tabula.technology/`.
6. Ito K, Patel PN, Gorham JM, McDonough B, DePalma SR, Adler EE, et al. Identification of pathogenic gene mutations in LMNA and MYBPC3 that alter RNA splicing. Proceedings of the National Academy of Sciences. 2017;114(29):7689–7694.
7. Ellingford JM, Thomas HB, Rowlands C, Arno G, Beaman G, Gomes-Silva B, et al. Functional and in-silico interrogation of rare genomic variants impacting RNA splicing for the diagnosis of genomic disorders. BioRxiv. 2019; p. 781088.
8. Palmisano A, Vural S, Zhao Y, Sonkin D. MutSpliceDB: A database of splice sites variants with RNA-seq based evidence on effects on splicing. Human Mutation. 2021;.
9. Ensembl. Ensembl REST API Version 13.1;. Available from: `https://rest.ensembl.org`.
10. NCBI. NCBI Nucleotide (nuccore);. Available from: `https://www.ncbi.nlm.nih.gov/nuccore`.
11. McRae J, Jaganathan K, Aswathnarayana S, Parry DA, Solli-Nowlan T. Illumina/SpliceAI; 2019. Available from: `https://github.com/Illumina/SpliceAI`.
